# The Grafting of Glands

**Published:** 1919-11-29

**Authors:** 


### THE GRAFTING OF GLANDS.

### A Record of French Experience.

Recently we expressed our criticism of the flights of English journalistic enterprise in connection with the prolongation of human life and the grafting of endocrine glands, in which it was far from our intention to cast doubt upon the value of the work performed in M. Voronoff's experimental laboratory, but simply to condemn the grossly misleading aspect which was given to the true medical bearing of such work in certain organs of the lay press. Still more recently we have received an extract from the *Gazette des Hôpitaux*, in which appears a report in which M. Voronoff communicated the result of certain experimental studies made at the *College de France* upon testicular grafts in animals. These are not only most interesting, but serve to throw considerably more light upon the actual nature of the work undertaken than did the reports we presumed to criticise.

### Testicular Grafts.

On this occasion, some hundred and twenty experiments were described upon goats, undertaken with a view to deciding whether a testicular graft could furnish an endocrine secretion. Grafts were made on twenty-five occasions with an entire testicle, fifty-eight times with a large portion of the gland and thirty-seven times with a small portion. In thirty-five cases these were placed under the skin, eighty-five times in bursæ, and twenty-three times under the peritoneum. In general outline the results showed that the peripheral portions of the grafts survived, while those more centrally placed degenerated, and that usually fragments of the gland grafted themselves better than the entire gland, but physiologically the effects produced were the same. As to position: it was in synovial cavities that the graft had the greatest chance of uniting successfully. Apparently the action is confined to male animals, and is extremely marked on both castrated and aged individuals. A particular experience is given in the case of one old male—upon which four fragments of normal testicle were grafted into the tunica vaginalis below the living testicle, whereupon the animal's vitality and character were rapidly transformed. Two months after the grafting, its sexual activity returned and procreation actually occurred. Later, the graft becoming absorbed, old age and accompanying impotence gradually returned. A further graft, however, united in the same way as the first and was followed by the same effects.

### Two Queries.

At the conclusion of this report, two questions were put to the author. First, if the testicular gland could be successfully grafted, how is it that a testicle deprived of its vessels, as so frequently occurs as the result of certain operative procedures, invariably necroses? Secondly, in what light did M. Voronoff consider the possibility of applying the results of these experiments to the human being? In the report to hand, the author replied to the first by repeating the fact experienced that a graft of the whole testicle united far more rarely than one consisting of a fragment, and, to the second, by saying that application to a man appeared to be not a little difficult of accomplishment, as it would never be easy to procure the graft, but there was the possibility that one might utilise the glands of the higher apes.

### The Conclusions Possible.

Thus the whole type of experiment and the value of actually *grafting* becomes far clearer, as do also the aims and ambition of the surgeon. In the first place, the results do not modify our views of the usual history of tissue grafts, which apparently here, as might be expected, gradually were absorbed, disappeared and became functionless. During this process, however, they undoubtedly imparted some endocrine secretion to the system which efficiently replaced that lost through atrophy of the original glands. Thus two points become clear. These experiments are valuable in establishing undeniably the direct connection between testicular endocrine secretion and male sexual activity, and if the corresponding procedure has been applied to the thyroid with equally definite results the corresponding connection may have been proved with regard to the metabolic activities of the body and its apparent general health. A proof, if one so considers it, of the rationality of endocrine therapy in certain clinical conditions. Such administration is relatively easy, but, as we recently pointed out, diagnosis of the exact and early states in which such endocrine treatments are advisable is *not* necessarily easy and certainly a subject to be treated with due caution by the average practitioner. Also as regards the lay-press claims of lengthened human life and "the return of youth," although a certain degree of success has been claimed in gland grafting it must be obvious that, as we remarked, the grafting is at best a very temporary process, and one which applied to the human being would be neither easy of accomplishment or pleasant in repetition at short intervals. Undoubtedly they are interesting and valuable experiments, but as they stand, appear to us, to offer no practical therapeutic advantage over the usual oral, hypodermic, or intravenous methods of endocrine administration, and certainly give no justifiable foundation for the misleading statements regarding the universal banishment of the signs of old age which were made by certain of the lay papers in this country.

We trust that M. Voronoff will take none of our comments as a biased criticism of his work, which is indeed most interesting and of great value, but the form in which such matters are placed before the lay public should, we think, be most carefully chosen.

